# Japanese Encephalitis in Hill and Mountain Districts, Nepal

**DOI:** 10.3201/eid1510.081641

**Published:** 2009-10

**Authors:** Anuj Bhattachan, Sumi Amatya, Tika Ram Sedai, Shyam Raj Upreti, Jeffrey Partridge

**Affiliations:** World Health Organization, Kathmandu, Nepal (A. Bhattachan, S. Amatya, T.R. Sedai, J. Partridge); and Ministry of Health and Population, Kathmandu (S.R. Upreti)

**Keywords:** Japanese encephalitis, epidemiology, endemic diseases, vaccination, viruses, letter

**To the Editor:** Nepal, a landlocked country in Southeast Asia with an estimated population of 27 million, is divided administratively into 5 regions; 75 districts comprise 3 ecological zones that run from east to west. Altitude increases from south to north: the 20-district Terai plains in the south, the hill region in the center with 39 districts, and the 16-district mountain regions in the north. Japanese encephalitis (JE) is seasonally endemic to the Terai region, which borders the northern India states of Uttar Pradesh and Bihar. The first outbreak of JE in Nepal was reported in 1978 from the Terai district of Rupendehi (*1*). Since then, JE infection has been reported in animal reservoirs and in humans throughout the Terai region (*1*–*5*). Although few publications describe the presence of JE outside the Terai, an outbreak of JE in Kathmandu valley in the hill region was confirmed in 1997 (*6*), and a 2006 study reported JE endemicity in Kathmandu Valley (*7*). In recent years, the Ministry of Health and Population in Nepal has introduced public health interventions, including mass immunization campaigns, for JE prevention in these known JE-endemic areas.

JE cases are captured through acute encephalitis syndrome (AES) surveillance conducted by the government of Nepal, with support from the World Health Organization (WHO), through a national sentinel surveillance network. From 2004 through 2006, a total of 108 laboratory-confirmed JE cases were reported from hill or mountain districts (excluding Kathmandu Valley). However, travel histories for case-patients were not available for these years to determine the origin of JE infection. We conducted a study to provide evidence of JE endemicity in hill and mountain districts of Nepal (excluding Kathmandu Valley).

Laboratory-confirmed JE case-patients identified in 2007 who reported residence in 1 of the 52 hill or mountain districts, excluding the 3 hill districts of the Kathmandu Valley, were followed up by surveillance medical officers. All patients (or next of kin if the patient was deceased or unavailable) were visited at home or contacted by telephone to confirm their residence and travel history during the 30 days before the onset of symptoms. Data and sample collection procedures and laboratory methods used for JE diagnosis were as previously reported by Partridge et al. (*7*). Patients were identified by the AES surveillance system if patients’ symptoms met the case definition for AES adopted from WHO guidelines (www.who.int/vaccines-documents/DocsPDF06/843.pdf), i.e., acute onset of fever and a change in mental status (e.g., confusion, disorientation, coma, or inability to talk); or if the patient experienced a new onset of seizures (excluding simple febrile seizures) or was identified as having AES, JE, or viral encephalitis. The study population included any person of any age who reported being a resident of 1 of the 52 hill or mountain districts (excluding Kathmandu Valley), who had been seen at any AES reporting site from January 1 through December 31, 2007, and who had been confirmed to have JE antibody by immunoglobulin M capture ELISA on a serum or cerebrospinal fluid (CSF) specimen.

In 2007, a total of 360 AES cases were reported from 40 hill or mountain districts. Of the 344 reported AES cases for which diagnostic samples were obtained, 90 (26%) were laboratory confirmed as JE from 21 hill and 3 mountain districts. Among laboratory confirmed JE cases, CSF samples were collected from 13 (14%) patients and serum samples from 77 (86%) patients (Table). The largest number of AES and laboratory-confirmed JE cases were reported during the monsoon months of August (23 cases) and September (33 cases). The higher proportions of cases in male patients and in those <15 years of age are consistent with the literature (*3*,*8*,*9*).

**Table:** Characteristics of Japanese encephalitis case-patients, hill and mountain districts (excluding Kathmandu Valley), Nepal, 2007

Characteristic	No. (%) case-patients
Age, y*	
<1	6 (7)
1–14	67 (74)
>15	71 (19)
Sex	
M	59 (66)
F	31 (34)
Death associated with Japanese encephalitis	5 (6)
Specimen type collected	
Serum	77 (86)
Cerebrospinal fluid	13 (14)
History of travel outside district of residence†	
No	84 (94)
Yes	3 (3)
Unknown	3 (3)

*Range 4 mo–79 y.           †During the 30 days before onset of symptoms.

Although most JE cases in Nepal occur in the lowland plains Terai region bordering India, our findings provide evidence that JE virus is also a cause of acute encephalitis in hill and mountain districts. This finding is consistent with the report of JE endemicity in the Kathmandu Valley, which is located in the hill region (*7*). Although the presence of both JE vectors and amplifying hosts has been confirmed in the hill region (*10*), additional studies are needed in high-altitude mountain districts to confirm the presence of environmental and ecological conditions that promote JE virus transmission. The national JE prevention and control program has focused on the Terai region and, more recently, on the Kathmandu Valley. However, results from our study suggest that Nepal may also have to consider the existence of JE in hill and mountain districts when developing future JE prevention and control programs. In addition, immunization against JE should be recommended for tourists to Nepal, even for those only planning to trek in the hills and mountains.

## References

[1] Bista MB, Shrestha JM. Epidemiological situation of Japanese encephalitis in Nepal. JNMA J Nepal Med Assoc. 2005;44:51–6. 16554872

[2] Pant GR, Lunt RA, Rootes CL, Daniels PW. Serological evidence for Japanese encephalitis and West Nile viruses in domestic animals of Nepal. Comp Immunol Microbiol Infect Dis. 2006;29:166–75. 10.1016/j.cimid.2006.03.003 16697904

[3] Akiba T, Osaka K, Tang S, Nakayama M, Yamamoto A, Kurane I, Analysis of Japanese encephalitis epidemic in Western Nepal in 1997. Epidemiol Infect. 2001;126:81–8. 11293685 PMC2869676

[4] Joshi D. Incidence of Japanese encephalitis in children: 1978, 1979, and 1980 outbreaks. NEPAS Journal. 1983;2:18–25.

[5] Wierzba TF, Ghimire P, Malla S, Banerjee MK, Shrestha S, Khanal B, Laboratory-based Japanese encephalitis surveillance in Nepal and the implications for a national immunization strategy. Am J Trop Med Hyg. 2008;78:1002–6. 18541784

[6] Zimmerman MD, Scott RM, Vaughn DW, Rajbhandari S, Nisalak A, Shrestha MP. Short report: an outbreak of Japanese encephalitis in Kathmandu, Nepal. Am J Trop Med Hyg. 1997;57:283–4. 9311637 10.4269/ajtmh.1997.57.283

[7] Partridge J, Ghimire P, Sedai T, Bista MB, Banerjee M. Endemic Japanese encephalitis in the Kathmandu Valley, Nepal. Am J Trop Med Hyg. 2007;77:1146–9. 18165538

[8] Vaughn DW, Hoke CH Jr. The epidemiology of Japanese encephalitis: prospects for prevention. Epidemiol Rev. 1992;14:197–221. 1337744 10.1093/oxfordjournals.epirev.a036087

[9] Darsie RF, Pradhan SP. The mosquitoes of Nepal: their identification, distribution and biology. J Am Mosq Control Assoc. 1990;22:69–130.

[10] Zimmerman MD, Scott RM, Vaughn DW, Rajbhandari S, Nisalak A, Shrestha MP. Short report: an outbreak of Japanese encephalitis in Kathmandu, Nepal. Am J Trop Med Hyg. 1997;57:283–4. 9311637 10.4269/ajtmh.1997.57.283

